# Supplementary figures and images for: Coronavirus surveillance in wildlife from two Congo basin countries detects RNA of multiple species circulating in bats and rodents
Source: PLoS One. 2021 Jun 9;16(6):e0236971. doi: 10.1371/journal.pone.0236971 (PMC8189465; doi:10.1371/journal.pone.0236971)

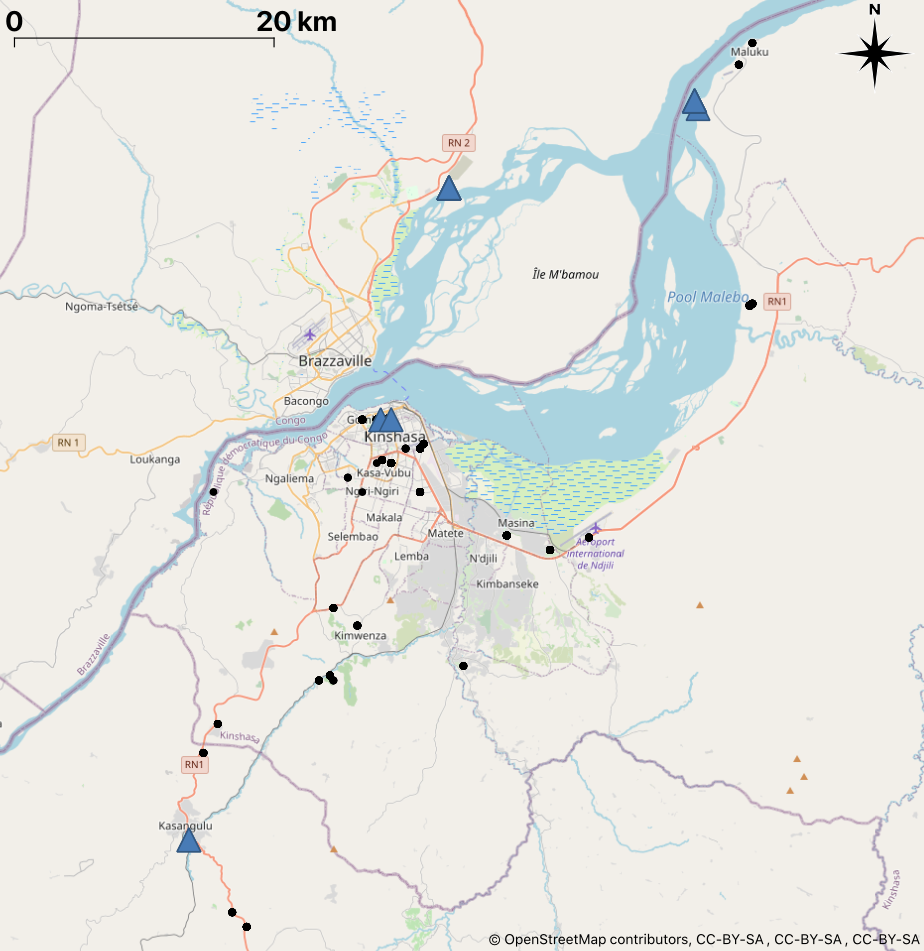

Supplement: S1 Fig — Geographical map indicating all sampling sites in and around the urban centers of Brazzaville and Kinshasa on either side of the Congo river, the border between the Republic of Congo and the Democratic Republic of the Congo. Locations where coronavirus RNA was detected in bats are highlighted with blue triangles, sampling sites without viral RNA detection are marked by black dots. Base map and data from OpenStreetMap and OpenStreetMap Foundation. (TIFF) [file pone.0236971.s001.tiff]

**A**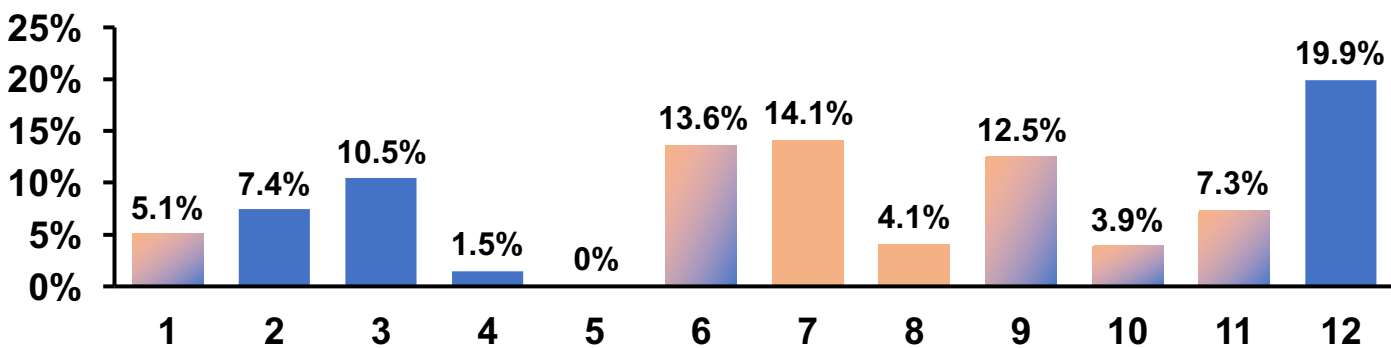**B**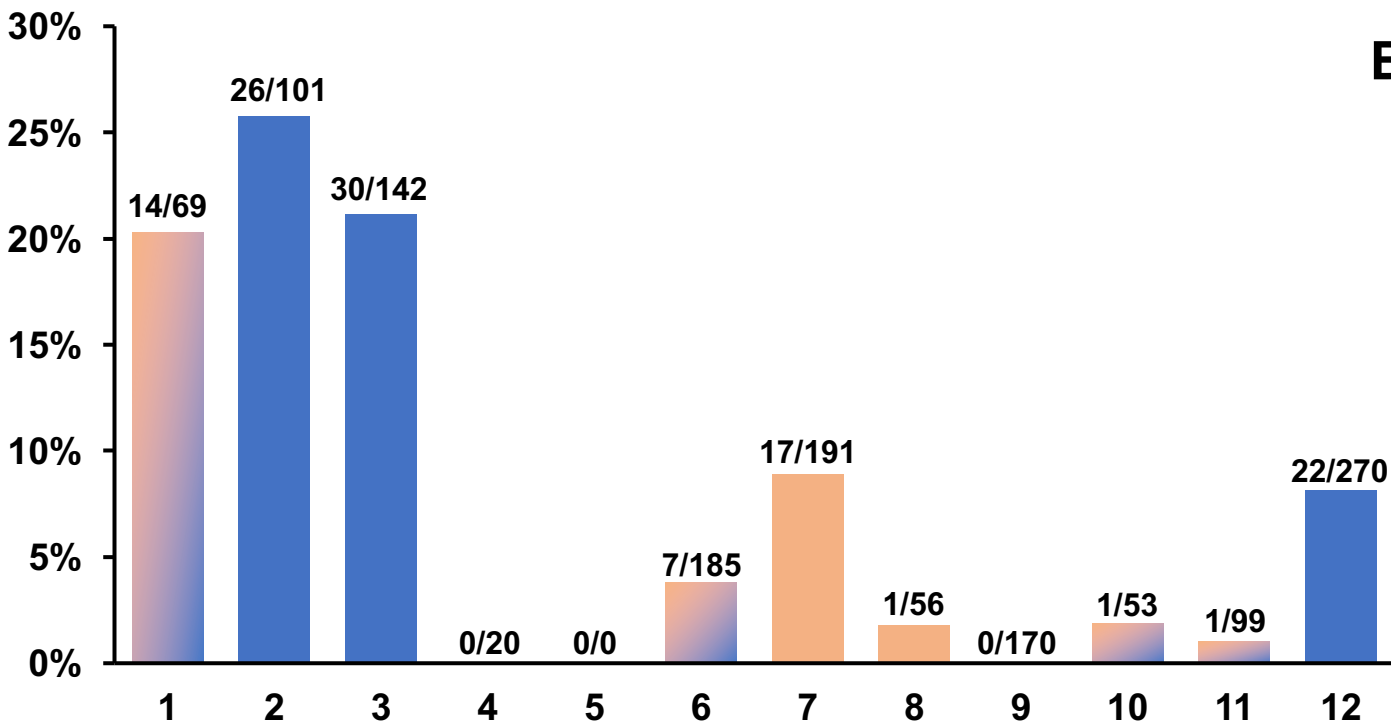**C**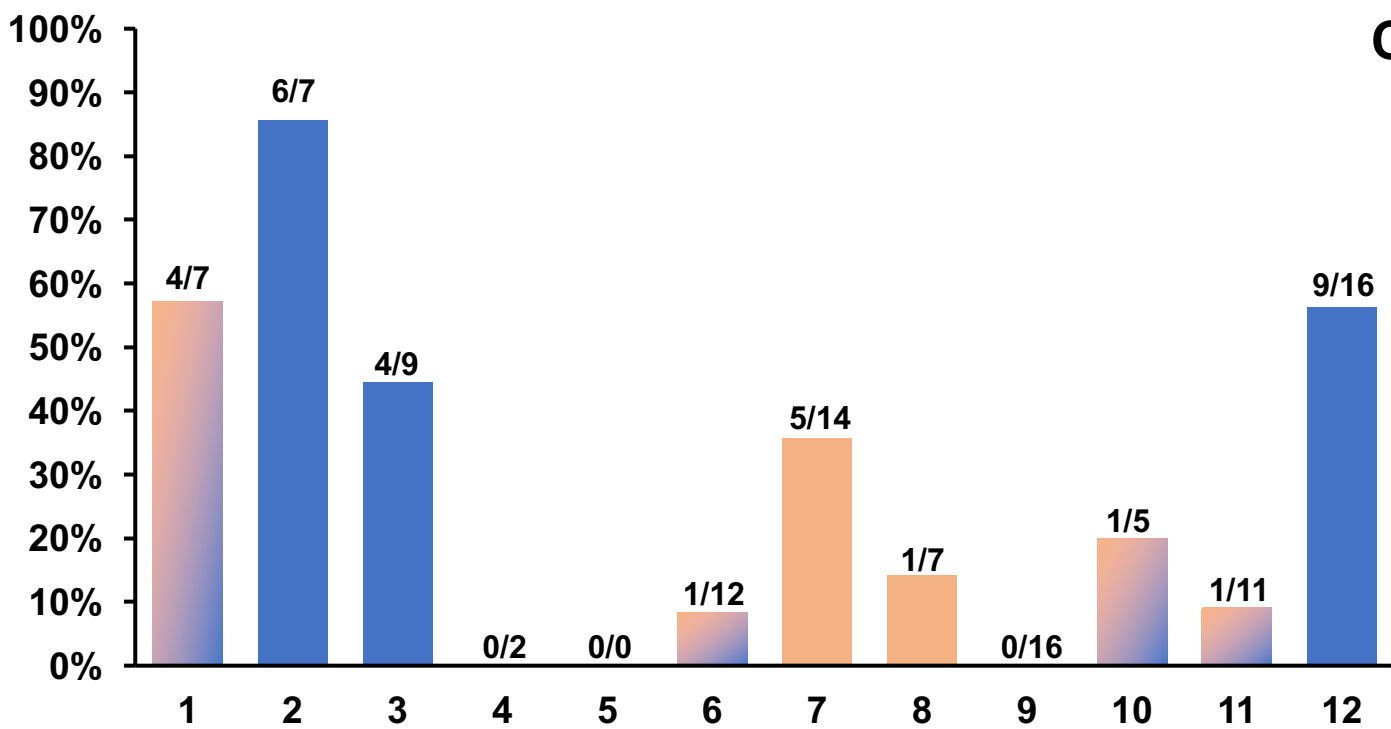

Supplement: S2 Fig — Bat sampling effort and detection rates by calendar month (cumulative over all years). Panel A is depicting the percentage of the total samples collected in each month, relative to total bat samples collected. Panel B depicts the percentage of coronavirus RNA positive animals relative to each month’s total samples, while panel C shows the percentage of sampling events with at least one coronavirus RNA detection per month. Blue bars are indicating that all samples were collected during local rainy season, while beige indicates the same for the local dry season. Gradient colored bars indicate months in which dry and wet season samples were collected depending on the location. (PDF) [file pone.0236971.s002.pdf]
