# Supplementary material for: Coronavirus surveillance in wildlife from two Congo basin countries detects RNA of multiple species circulating in bats and rodents
Source: PLoS One. 2021 Jun 9;16(6):e0236971. doi: 10.1371/journal.pone.0236971 (PMC8189465; doi:10.1371/journal.pone.0236971)
Supplement: S3 Table — Geographical and temporal distance between sampling events with detections of identical or closely related (>95% nucleotide identities) coronavirus RNA sequences (compare also S1 and S2 Tables). (DOCX) [file pone.0236971.s005.docx]

S3 Table: Geographical and temporal distance between sampling events with detections of identical or closely related (>95% nucleotide identities) coronavirus RNA sequences (compare also Supplements 1 & 2)

A) Coronavirus cluster Q-/W-Beta-1

|  | DRC-C | DRC-E | ROC-H |
| --- | --- | --- | --- |
| DRC-C | X | 1169 km | 526 km |
| DRC-E | 28 days | X | 1330 km |
| ROC-H | 941 days | 913 days | X |

B) Coronavirus cluster Q-/W-Beta-2

|  | DRC-A | DRC-B | DRC-G | DRC-H | DRC-I | DRC-J | DRC-K | DRC-L | DRC-M | DRC-Q | ROC-B | ROC-D | ROC-F | ROC-G |
| --- | --- | --- | --- | --- | --- | --- | --- | --- | --- | --- | --- | --- | --- | --- |
| DRC -A | X | 34 km | 317 km | 319 km | 379 km | 374 km | 1567 km | 287 km | 423 km | 0 km | 646 km | 665 km | 655 km | 18 km |
| DRC -B | 58 days | X | 283 km | 285 km | 346 km | 342 km | 1559 km | 320 km | 389 km | 34 km | 619 km | 639 km | 631 km | 20 km |
| DRC -G | 475 days | 417 days | X | 10 km | 66 km | 85 km | 1476 km | 602 km | 107 km | 317 km | 482 km | 506 km | 524 km | 303 km |
| DRC -H | 476 day | 418 days | 1 day | X | 62 km | 77 km | 1466 km | 604 km | 108 km | 318 km | 491 km | 515 km | 533 km | 305 km |
| DRC -I | 480 days | 422 days | 5 days | 4 days | X | 45 km | 1440 km | 665 km | 64 km | 379 km | 494 km | 518 km | 543 km | 366 km |
| DRC -J | 485 days | 427 days | 10 days | 9 days | 5 days | X | 1399 km | 661 km | 105 km | 374 km | 538 km | 563 km | 587 km | 362 km |
| DRC -K | 510 days | 452 days | 35 days | 34 days | 30 days | 25 days | X | 1742 km | 1480 km | 1566 km | 1917 km | 1941 km | 1975 km | 1572 km |
| DRC -L | 1030 days | 972 days | 555 days | 554 days | 550 days | 545 days | 520 days | X | 705 km | 288 km | 837 km | 851 km | 824 km | 300 km |
| DRC -M | 1216 days | 1158 days | 741 days | 740 days | 736 days | 731 days | 706 days | 186 days | X | 423 km | 443 km | 467 km | 497 km | 408 km |
| DRC -Q | 1708 days | 1650 days | 1233 days | 1232 days | 1228 days | 1223 days | 1198 days | 678 days | 492 days | X | 646 km | 665 km | 655 km | 18 km |
| ROC -B | 456 days | 398 days | 19 days | 20 days | 24 days | 29 days | 54 days | 574 days | 760 days | 1252 days | X | 24 km | 72 km | 628 km |
| ROC -D | 509 days | 451 days | 34 days | 33 days | 29 days | 24 days | 1 day | 521 days | 707 days | 1199 days | 53 days | X | 59 km | 647 km |
| ROC -F | 513 days | 455 days | 38 days | 37 days | 33 days | 28 days | 3 days | 517 days | 703 days | 1195 days | 57 days | 4 days | X | 637 km |
| ROC -G | 1145 days | 1087 days | 670 days | 669 days | 665 days | 660 days | 635 days | 115 days | 71 days | 563 days | 689 days | 636 days | 632 days | X |

C) Coronavirus cluster Q-/W-Beta-3

|  | DRC-C | DRC-D | DRC-O | DRC-P | ROC-C | ROC-D | ROC-E | ROC-F |
| --- | --- | --- | --- | --- | --- | --- | --- | --- |
| DRC-C | X | 2  km | 433  km | 433  km | 909  km | 909  km | 910  km | 915  km |
| DRC-D | 1  day | X | 434  km | 434  km | 911  km | 911  km | 912  km | 917  km |
| DRC-O | 1096 days | 1095 days | X | 0  km | 836  km | 836  km | 814  km | 813  km |
| DRC-P | 1100 days | 1099 days | 4  days | X | 836  km | 836  km | 814  km | 813  km |
| ROC-C | 38  days | 37  days | 1058 days | 1062 days | X | 0  km | 46  km | 59  km |
| ROC-D | 87  days | 86  days | 1009 days | 1013 days | 49  days | X | 46  km | 59  km |
| ROC-E | 89  days | 88  days | 1007 days | 1011 days | 51  days | 2  days | X | 13  km |
| ROC-F | 91  days | 90  days | 1005 days | 1009 days | 53  days | 4  days | 2  days | X |
