# Supplementary material for: Coronavirus surveillance in wildlife from two Congo basin countries detects RNA of multiple species circulating in bats and rodents
Source: PLoS One. 2021 Jun 9;16(6):e0236971. doi: 10.1371/journal.pone.0236971 (PMC8189465; doi:10.1371/journal.pone.0236971)
Supplement: Attachment — Submitted filename: Response_to_Reviewers_.docx [file pone.0236971.s006.docx]

Response to reviewers

****All line references based on the revised manuscript version WITHOUT track changes ****

| Editorial |  |
| --- | --- |
| 1. Please ensure that your manuscript meets PLOS ONE's style requirements, including those for file naming. | The manuscript has been updated to meet the requirements. |
| 2. In your Methods section, please provide additional location information of the collection sites, including geographic coordinates for the data set if available. | The geographic coordinates of the collection sites for all samples are listed in Supplement 2. We included reference to that in the Methods section (lines 122-123). |
| 3. Your ethics statement should only appear in the Methods section of your manuscript. If your ethics statement is written in any section besides the Methods, please move it to the Methods section and delete it from any other section. Please ensure that your ethics statement is included in your manuscript, as the ethics statement entered into the online submission form will not be published alongside your manuscript. | We have moved the ethics statement into the Methods section (lines 152-157). |
| 4. We note that Figure 1 and Supplement_1 file in your submission contain map  images which may be copyrighted. All PLOS content is published under the Creative Commons Attribution License (CC BY 4.0), which means that the manuscript, images, and Supporting Information files will be freely available online, and any third party is permitted to access, download, copy, distribute, and use these materials in any way, even commercially, with proper attribution. For these reasons, we cannot publish previously copyrighted maps or satellite images created using proprietary data, such as Google software (Google Maps, Street View, and Earth). For more information, see our copyright guidelines: [http://journals.plos.org/plosone/s/licenses-and-copyright](https://deref-gmx.net/mail/client/ws7UCfaObDo/dereferrer/?redirectUrl=http%3A%2F%2Fjournals.plos.org%2Fplosone%2Fs%2Flicenses-and-copyright).  We require you to either (1) present written permission from the copyright holder to publish these figures specifically under the CC BY 4.0 license, or (2) remove the figures from your submission: | We replaced the original Figure 1 and Supplement 1 with maps based on OpenStreetMaps (CC-BY-SA). |
| 5. Please include captions for your Supporting Information files at the end of your manuscript, and update any in-text citations to match accordingly. Please see our Supporting Information guidelines for more information: http://journals.plos.org/plosone/s/supporting-information. | Included and cited accordingly. |
| 6. Thank you for stating the following in the Financial Disclosure section:  "NW and TG received awards GHN-A-OO-09-00010-00 and AID-OAA-A-14-00102 from the United States Agency for International Development ([https://www.usaid.gov](https://deref-gmx.net/mail/client/Nou4RFywa2Q/dereferrer/?redirectUrl=https%3A%2F%2Fwww.usaid.gov)). The funders had no role in study design, data collection and analysis, decision to publish, or preparation of the manuscript."  We note that one or more of the authors are employed by a commercial company: 'Metabiota Inc', 'Labyringth Global Health', 'Development Alternatives, Inc.', 'Mosaic, Cameroon', 'Etiologic, Oakland' and 'Pinpoint Science, San Francisco'.  (1) Please provide an amended Funding Statement declaring this commercial affiliation, as well as a statement regarding the Role of Funders in your study. If the funding organization did not play a role in the study design, data collection and analysis, decision to publish, or preparation of the manuscript and only provided financial support in the form of authors' salaries and/or research materials, please review your statements relating to the author contributions, and ensure you have specifically and accurately indicated the role(s) that these authors had in your study. You can update author roles in the Author Contributions section of the online submission form.  Please also include the following statement within your amended Funding Statement.  “The funder provided support in the form of salaries for authors [insert relevant initials], but did not have any additional role in the study design, data collection and analysis, decision to publish, or preparation of the manuscript. The specific roles of these authors are articulated in the ‘author contributions’ section.”  If your commercial affiliation did play a role in your study, please state and explain this role within your updated Funding Statement.    (2) Please also provide an updated Competing Interests Statement declaring this commercial affiliation along with any other relevant declarations relating to employment, consultancy, patents, products in development, or marketed products, etc.  Within your Competing Interests Statement, please confirm that this commercial affiliation does not alter your adherence to all PLOS ONE policies on sharing data and materials by including the following statement: "This does not alter our adherence to  PLOS ONE policies on sharing data and materials.” (as detailed online in our guide for authors http://journals.plos.org/plosone/s/competing-interests) . If this adherence statement is not accurate and  there are restrictions on sharing of data and/or materials, please state these. Please note that we cannot proceed with consideration of your article until this information has been declared.  Please include both an updated Funding Statement and Competing Interests Statement in your cover letter. We will change the online submission form on your behalf.  Please know it is PLOS ONE policy for corresponding authors to declare, on behalf of all authors, all potential competing interests for the purposes of transparency. PLOS defines a competing interest as anything that interferes with, or could reasonably be perceived as interfering with, the full and objective presentation, peer review, editorial decision-making, or publication of research or non-research articles submitted to one of the journals. Competing interests can be financial or non-financial, professional, or personal. Competing interests can arise in relationship to an organization or another person. Please follow this link to our website for more details on competing interests: http://journals.plos.org/plosone/s/competing-interests | We included an updated Funding Statement and an updated Competing Interests Statement in the cover letter. |
| Reviewer #1: | - |
| The paper reports on the surveillance of coronaviruses among bats, rodents, shrews and non-human primates in the congo basin. The study was performed across 12 years and opportunistically sampled 3561 animals for coronavirus RNA using two separate assays. Nearly all of the positives were from bats with the additional detection of two rodent positives. Based on the results the authors also performed some analyses to correlate detection of positives with observed ecological features such as seasonality. The paper is well written and the conclusions are thoughtful and well-reasoned. | - |
| My main request is the need to better details on the sampling methodology of the animals sampled- specifically in terms of time and frequency of sampling and repeat sampling at the same site if performed. This can be generally included in the text or better illustrated in one of the figures. | We added more details about the sampling approach in the Materials and Methods section (lines 129-140) |
| The authors report that they identified no coronaviruses from non-human primates or shrews and only two from rodents. They comment that CoVs in wildlife are scarcer than in bats, and this may be true but consider the fragmented opportunistic sampling generally dedicated to sampling of “non-bats” for coronaviruses. It is difficult to discern from the way the data is reported in the manuscript or even from the supplementary table 2 how dedicated or opportunistic the sampling has been for the non-human primates across the 836 individuals from the total sampling timespan. Were some of the animals sampled more than once? If so, it would be important to highlight in text (that dedicated and repeat sampling was conducted). | Repeat sampling did not take place for non-human primates. We have expanded the section on sample collection for more clarity (lines 129-140). |
| My second concern is regarding the two assays used in manuscript. Supp 2 indicates a real time assay used for some samples – though this was not indicated in text- can the authors please add or clarify this? Also can the authors please clarify if the Quan assay was modified from the original? were the primers optimized? The Watanabe assay was modified as indicated. Can the authors comment on the sensitivity between the assays? Or what controls were used for the assays? The reason for my query is that these assays don’t amplify the same gene region of the genome (of course) and so the sequences cannot be directly compared so it might be a little difficult to determine if the two assays identified sequences from the same or different viruses. It seems that it was somewhat frequent that one assay was capable of detecting sequences missed by the other assay. What was the frequency of overlap that the assays were capable of detecting the same ‘virus’ vs. the frequency that the assays missed or detected very different viruses? | - A qPCR was indeed performed in the case of 4 bonobos. However, since they were negative (as all tested primates) and the animals were also tested with the other assays we excluded this information, to avoid confusion.  - We have added a clarification regarding the Quan assay (not modified; compare lines 171-177).  - No sensitivity testing was performed, as that would only be informative for the tested viruses, but not others (such as novel ones)  - A clarification with regards to the control was added (lines 184-186).  - We elaborated on the issue of differences in detection in the text. In brief: In 33 cases both assays detected CoV RNA in the same bat. In 31 of them we regard it as the same virus (compare lines 237-243 and 348-353). |
| Then, did the authors try combining the two assays on the positives to amplify the sequence region between primer regions in order to amplify both a longer gene region but also confirm identification of the same viral sequences with the different assays? Is one assay better at detecting a specific clade of coronavirus? As this was the first report of the rodent coronaviruses, full genomes would have been more informative. | A long range PCR linking both PCR target regions was not attempted, as only in about a quarter of the cases both PCRs amplified viral RNA. We do not see a difference in the detection of alpha- and betacoronaviruses, but it is fair to assume, that such a difference with regards to certain coronaviruses / Species / Subgenera exists. However we do not consider the data sufficient to draw definite conclusions about that (compare lines 237-243 and 348-353). |
| I have no specific line issues except that the authors can consider using ‘alphacoronaviruses’ or ‘betacoronaviruses’ instead of Alpha coronaviruses or Beta coronaviruses (line 334 and 308). | This has been changed accordingly throughout the manuscript. |
| Can the authors define the term wet season? Dry season? Exactly which months does this include? | The definition we used for wet and dry season has been included in the methods section (lines 219-221). |
| Similarly, in supplement 4 – which months are indicated by 1-12? Which years? | The legend has been updated for clarification (line 617). |
| Its difficult to determine how the sequences were named in the study – please consider the naming convention suggested by the Coronavirus study group: The species Severe acute respiratory syndrome related coronavirus: classifying 2019-nCoV and naming it SARS-CoV-2 (page 4). Using a method that names sequences based on most related sequences as has previously been done creates significant confusion. | Sequence/isolate “names” were assigned by the PREDICT consortium consecutively throughout the duration of the project. They are associated with the GenBank files, but to simplify things we do not mention any of them in the manuscript. We use “cluster names” in the phylogenetic trees and animal IDs where more specificity seemed necessary. |
| Please consider adding sequence identifiers to the sequences in the phylogenies? Like HKU8 to bat sequence Miniopterus from China etc. It also helps to orient the reader in the tree and relate it to previously reported coronaviruses (and species). | We did consider that, and made some updates. However, the naming for the bat CoV sequences we included here as “references” is highly inconsistent. Since we prefer the attention to be on the host and from where (country) the sequence was obtained, and in the interest of readability, we decided to stick with just the GenBank accession numbers for bat-CoV identification in the trees. |
| Moreover, it is challenging to navigate between the trees so determine if similar coronaviruses were detected with the different assays, as the same reference sequences are not present in all trees- is this due to the regions availability to compare? (particularly between the 229E and NL63 clades). | That is indeed the case. We selected a “base set” of CoVs for which full genomes are available to ensure the two trees have the same basic structure, but then included additional partial sequences that showed highest identities with the ones we detected. |
| Reviewer #2: | - |
| General Comment : | - |
| The study provides new information on the diversity of coronavirus species circulating in the Democratic Republic of Congo and the Republic of Congo. However, at any time, this study does not refer to a recent published study on coronaviruses conducted in Gabon, a country in the Congo Basin. | The manuscript has been modified and our results are now also discussed in the context of the recent findings from Gabon (lines 106-109, 364-367, 413-417, 425-427, and 446-449). |
| In addition, full-length genomes with particularly spike gene at least will increase the importance of the work. | This would indeed be desirable, but is not possible in the near future. Project funds ran out in 2020, local recourses and capabilities for this kind of task a currently not available, and DRC (where most samples are stored) has put a moratorium on the export of any samples/nucleic acid since the onset of the COVID-19 pandemic. |
| Specific comments : |  |
| Page 1, lines 1-2 : As the Congo Basin is not limited to these two countries, I propose that the title be more precise. For example « Coronavirus surveillance in two countries in Congo basin wildlife detects … ». | The title was changed along those lines (lines 1-2) |
| Page 4, lines 91-94 : The authors could also refer to the study by Maganga et al. (Maganga GD, Pinto A, Mombo IM, et al. 2020. Scientific Reports. 10:7314, 10.1038/s41598-020-64159-1) carried out in a Congo Basin country. | The reference was added accordingly (lines 106-109). |
| Page 5 Line 102 : To determine the degree of coronavirus circulation, a serological study would be more appropriate, therefore I suggest this sentence : « Our goal was to determine the diversity of circulating coronaviruses, using… ». | We updated the sentence accordingly (line 117). |
| Line 111 : The authors state « Fecal samples were collected from free-ranging NHPs ». Can you explain how the collection was done? | We added a respective reference to the methods section (line 127, Seimon et al. 2015). |
| Pages 5-6 Lines 120-121 : Why extraction with Trizol in addition to extraction using commercial kits? And why different extraction kits for swabs collected prior to and after 2014? | Some of the techniques and protocols changed during the very long duration of the project. The switch to sample collection in Trizol reflects an increased precaution, as it inactivates potentially infectious and zoonotic viruses. Some of this is now discussed in lines 338-342. |
| Lines 124-138 : The authors used two different PCR assays that amplify the same gene (RdRp), to broaden the detection spectrum of coronaviruses regardless of the animal host. However, the sizes of the amplified fragments are quite short for a highly conserved gene such as RdRp. It would be relevant to obtain sequences of a gene of interest such as the Spike gene if complete genomes cannot be obtained. | Obtaining additional sequence of for example Spike would indeed be desirable, but is not possible in the near future. Project funds ran out in 2020, local recourses and capabilities for this kind of task a currently not available, and DRC (where most samples are stored) has put a moratorium on the export of any samples/nucleic acid since the onset of the COVID-19 pandemic. |
| Page 7 Line 172 : The collection period should be indicated in the Materials and Methods section. | This has been moved from the Results to the Materials and Methods section accordingly (lines 122-123). |
| Line 177 : The authors state “others (36)“ as tissue sample. Can you specify the nature of these samples ? | This has been updated and specified in the text (lines 228-229). |
| Lines 178-179 : Authors should be careful not to create confusion between samples and animals. The authors indicate that 121 animals were detected positive, corresponding to 23 oral swabs, 102 rectal swabs and 1 pool of liver and spleen. However, for the 83 and 73 samples in which viral RNA was found, can we know how many individuals these numbers correspond to? | The two respective sentences were rewritten for more clarity on this issue (lines 229-231 and 237-238). |
| Were the 73 samples that tested positive using Quan PCR assay the same as the 83 samples that tested positive using Watanabe PCR assay ? If so, this should be specified. | We have modified the respective sentence/statement for more clarity (lines 237-238). |
| Can the authors explain why the search for coronaviruses was done in the liver and spleen? These are not organs that are conventionally screened for these viruses. | We included a sentence in the methods and some more details on that matter in the results and discussion (lines 143-145, 231-236, and 342-347). |
| Page 8 Lines 187-190 : As was done for rodents, indicate for bats, in brackets, the number of positive individuals out of the total number tested. | The section has been updated accordingly (lines 251-256). |
| At any time in the materials and methods section there was no mention of how the identification of the species was done. | A respective statement including references has been included in the Materials and Methods section (lines 127 and 141-143). |
| Lines 194-195 : In the introduction, I suggest that the classification of bats be briefly presented so that the terms Yinpterochiroptera and Yangochiroptera do not appear abruptly in the results section. Also, write throughout the document Yinpterochiroptera instead of Yinpterchiroptera. And write them in italics throughout the document. | The suggested updates have been made (lines 80-82 and throughout the manuscript). |
| Page 10 Lines 243-246 : The authors should discuss further the seasonal difference in positivity rates. For example, what factors related to bats might explain them? Maganga et al. 2020 had, for the species studied, identified the rainy season as the birthing season, which may justify a higher viral shedding rate. | We have expanded our discussion on that issue (lines 364-367). |
| Table 1 : Correct Yinpterochiroptera | corrected |
| Figure 2 and 3: I suggest the authors to add sequences of Alphacoronavirus of bats from Gabon and to include some sequences of Betacoronavirus of bats from this area. Especially since Figure 2 seems to show that the same viruses would circulate in Gabon and the Republic of Congo (See the first cluster of the phylogenetic tree). Moreover, the authors do not even discuss this observation. | We included sequences of Gabon Bat CoVs in the respective analysis and expanded our discussion on the coronaviruses in Central Africa (Figure 2, lines 413-417, 425-427, and 446-449). |
